# Evidence for a severe cognitive subgroup in a comprehensive neuropsychological Post-COVID-19 syndrome classification

**DOI:** 10.1038/s41598-025-25453-y

**Published:** 2025-11-18

**Authors:** Luisa T. Balz, Deborah K. Erhart, Ingo Uttner, Dorothée E. Lulé, Hayrettin Tumani

**Affiliations:** Department of Neurology, Faculty of Medicine, Ulm University Oberer Eselsberg, 45-89071 Ulm, Germany

**Keywords:** Post-COVID-19 syndrome, Neuropsychological tests, Mood disorders, Fatigue, Cluster analysis, Diseases, Health care, Neurology, Neuroscience, Psychology, Psychology

## Abstract

Approximately 7% of adults develop Post-COVID-19 syndrome (PCS). Cognitive impairment is common, but its profile in PCS is not well defined and requires clearer differentiation from general (post-)illness effects. The aim of this study was to identify distinct cognitive profiles in PCS and to characterize them with respect to broader psychological factors such as fatigue, depression, anxiety, and personality. To this end, we assessed cognition, fatigue, depression, anxiety, and personality traits in PCS patients, convalescents (CV), healthy controls (HC), and post-viral syndrome patients (PV) of different etiology. Cognitive performance was assessed with an extensive protocol covering multiple cognitive domains, including verbal and nonverbal short-term memory, verbal and nonverbal working memory, verbal and nonverbal episodic memory, visuoconstruction, attentional functions, and executive functions, alongside patient-reported measures of fatigue, depression, anxiety, and personality, as well as self-reported information on lifetime psychiatric diagnoses. Group comparisons and discriminant analysis (*p* = .065) identified the tests that best differentiate the groups, while exploratory cluster analysis allowed for data-driven group assignment. In this study, *N* = 54 PCS, *N* = 42 HC, *N* = 25 CV, and *N* = 12 PV were included. Patients with PCS show low mean scores and cluster into two subgroups: 7.5% with severe objective cognitive impairments (PCS_SCI_) and 92.5% with cognitive performance comparable to CV, HC, and PV (Mixed_(non-SCI)_). The PCS_SCI_ subgroup showed pronounced objective impairments, particularly in attentional, memory, and executive domains. Across the entire PCS sample, objective cognitive impairments were not predicted by fatigue severity or affective state (depression or anxiety), although lifetime psychiatric disorders and cognitive fatigue were significantly more prevalent in PCS_SCI_. Among patients with PCS, a distinct subgroup of individuals presents with severe cognitive deficits, significant cognitive fatigue, and an increased lifetime vulnerability for psychiatric disorders, highlighting the need for targeted diagnostic assessments and personalized (psycho-)therapeutic interventions.

**Supplementary Information:**

The online version contains supplementary material available at 10.1038/s41598-025-25453-y.

## Introduction

Post-COVID-19 syndrome (PCS) is defined by persistent post-illness complaints, including both physical and cognitive symptoms, following acute SARS-CoV-2 infection for longer than 12 weeks, which cannot be explained by an alternative diagnosis^1^. Among these symptoms, cognitive impairment, often referred to as “brain fog”, is one of the most commonly reported, affecting particularly attention, working memory, cognitive processing speed, and verbal fluency^2–4^. Approximately 50% of Post-COVID-19 patients exhibit cognitive dysfunction, when using screening tools (e.g., MoCA or MMSE) for cognition^4–6^. When using detailed neuropsychological testing, numbers may range between 3.2% and 24% in PCS patients within 1 year after infection^7,8^. Despite the increased psychological distress inflicted by PCS and depressive symptoms occurring in approximately 30% to 45% of cases^3,5,9^, cognitive dysfunction may occur independently of depression severity^5,10^ and cannot be attributed to distress alone, highlighting the need for a more nuanced understanding of its underlying mechanisms^3^. Further, COVID-19 is closely linked to cognitive and motor fatigue similar to myalgic encephalomyelitis or chronic fatigue syndrome in up to 82% of patients^3,5,9^, but the relationship between affective state (depression and anxiety), fatigue and cognitive impairment remains unclear. A central challenge in PCS research is the discrepancy between subjective cognitive complaints and objective cognitive markers. While some individuals with self-reported cognitive difficulties exhibit measurable cognitive deficits, others do not, indicating that subjective reports do not always align with neuropsychological assessments^11–14^. This inconsistency raises questions about the extent to which cognitive impairment in PCS is driven by neurobiological dysfunction versus psychological factors such as affective state, or fatigue^12,15,16^.

Accumulating evidence also supports the assumption that SARS-CoV-2 infection may exacerbate preexisting cognitive dysfunction^7,17^. On the other hand, it is also a matter of discussion whether increased premorbid vulnerability to psychiatric disorders, possibly exacerbated by specific personality traits such as neuroticism or low conscientiousness, may aggravate the risk of PCS patients to develop severe cognitive impairment^18,19^.

For a better understanding of the complex nature of cognitive deficits in PCS, we assessed cognition as well as fatigue, symptoms of depression and anxiety, and personality traits in PCS patients with subjective reports on cognitive impairment, compared to post-viral conditions of different etiology and varying reported disease impact. With respect to the findings of Wulf et al.^8^, who identified a small subgroup of PCS patients with objectively measurable cognitive impairments, our study is not limited to statements about the performance level of the PCS group as a whole, as in most previous studies, but also aims to focus on the individual performance level. By identifying more precise subgroups of PCS patients based on individual neuropsychological performance patterns, we aim to capture the heterogeneity of cognitive outcomes within PCS and to explore potential mechanisms connecting cognitive, affective, and personality-related factors. This approach could offer clinically relevant insights for targeted diagnostics and personalized (psycho-)therapeutic strategies.

## Materials and methods

### Design and participants

Fifty-four PCS, twelve Post-Viral (PV), and twenty-five Convalescent (CV) patients were recruited from Ulm University’s Neurology Department, along with forty-two age-, sex-, and education-matched Healthy Controls (HC). All participants gave written informed consent prior to their inclusion in the study, and the study was conducted in accordance with the Declaration of Helsinki and approved by Ulm University’s ethics committee (No. 16/23). PCS and PV patients were recruited from the Post-COVID-19 outpatient unit, while CV individuals came through the neurological emergency department. Participants were consecutively enrolled in a prospective cross-sectional study (03/2023–11/2024). HC were recruited via advertisements in sports facilities and public locations. PCS, CV, and HC subjects were all ≥ 12 weeks after COVID-19 and had a confirmed positive antigen rapid test during the acute phase. PCS patients were diagnosed according to the Delphi consensus criteria for Post-COVID-19 Syndrome^20^ and additionally required a confirmed positive PCR test (Polymerase Chain Reaction) for SARS-CoV-2^1^. CV participants had a confirmed COVID-19 infection but never developed PCS; they experienced temporary, non-specific physical symptoms after recovery that were not caused by COVID-19, and neurological or other relevant diagnoses were ruled out. HC also had an acute COVID-19 infection but fully recovered without any lingering symptoms. PV subjects had a viral infection of different etiology (e.g., Epstein-Barr virus infection), documented by medical records. For PV cases before 2020, we assumed SARS-CoV-2 viruses did not significantly contribute among circulating cold viruses. For PV cases after 2020, either rapid antigen testing and/or PCR at the time of acute infection was negative for SARS-CoV-2. Exclusion criteria included motor, speech, or language impairments affecting test validity, severe psychiatric disorders (e.g., schizophrenia or psychosis), and neurological or medical conditions known to impact neuropsychological functioning (e.g., stroke or previously diagnosed neurocognitive disorder).

## Procedure

All subjects were screened by a trained physician for major physical or psychiatric disorders, with PCS, PV, and CV participants also receiving a detailed neurological examination. All subjects performed an extensive neuropsychological assessment targeting memory, attention, executive, and visuospatial functions. In addition, participants reported outcome measures on depression, anxiety, and fatigue, while demographics and clinical data were collected via semi-structured interviews.

## Neuropsychological assessment

The neuropsychological assessment comprised validated instruments, targeting various cognitive domains. Verbal short-term and working memory were evaluated using the Digit Span Test from the Wechsler Memory Scale-Revised (WMS-R)^21^, while nonverbal short-term and working memory were assessed with the Block-Tapping Test (WMS-R). Verbal episodic memory was measured with the Verbal Learning and Memory Test (VLMT)^22^, and nonverbal episodic memory was assessed using the Rey-Osterrieth Complex Figure Test^23^. Attention domains, including alertness, divided attention, and incompatibility, were evaluated with the German Testbatterie zur Aufmerksamkeitsprüfung (TAP)^24^. The Symbol Digit Modalities Test (SDMT)^25^ was employed to measure information processing speed as well as divided and selective attention, and verbal fluency was assessed with the Regensburger Wortflüssigkeitstest (RWT), the German adaptation of verbal fluency measures^26^.

## Affective state and fatigue

Lifetime mental disorders were recorded based on patient reports of pre-existing, clinically diagnosed psychiatric conditions (e.g. depression, anxiety disorders or PTSD), which had been diagnosed by a psychiatrist or psychotherapist before the start of the COVID-19 pandemic.

Symptoms of depression were assessed using the well-established Patient Health Questionnaire-9 (PHQ-9)^27^. The total score ranges from 0 to 27, with ≥ 10 indicating potential major depression. Severity is classified as mild (≥ 10), moderate (≥ 15), and severe (≥ 20). Symptoms of anxiety were assessed using the Generalized Anxiety Disorder 2-item (GAD-2), with a score range of 0 to 6^28^. A threshold of 3 points serves as the recommended cut-off, indicating potential cases where further diagnostic evaluation for generalized anxiety disorder is advised.

Fatigue was measured using the Fatigue Scale for Motor and Cognitive Functions (FSMC), a 20-item questionnaire evaluating motor and cognitive fatigue^29^. Each item is rated on a 5-point Likert scale (1–5), with cut-offs for motor fatigue: mild (≥ 22), moderate (≥ 27), severe (≥ 32); for cognitive fatigue: mild (≥ 22), moderate (≥ 28), severe (≥ 34).

## Personality traits

Using the NEO Five-Factor Inventory (NEO-FFI 30), the Big Five personality traits were assessed: Neuroticism, Extraversion, Openness to Experience, Agreeableness, and Conscientiousness^30^. The NEO-FFI consists of 30 items, with each of the five factors being measured by six items.

### Statistical analysis

Statistical analyses were conducted using SPSS 29 and R. A priori power calculations, based on PCS and a combined HC/CV cohort, ensured 80% power to detect medium to large effect sizes (partial *η²*=0.06–0.14) with *α* = 0.05, requiring *N* = 50 PCS and *N* = 50 HC/CV. Post hoc, HC was subdivided into HC and CV to account for individuals who experienced COVID-19 with non-specific physical symptoms but did not meet the PCS criteria, allowing for the assessment of potential subtle cognitive effects. Additionally, *N* = 12 PV patients were recruited but not included in the initial power calculations. Gender matching was assessed using chi-square tests. Group differences in cognition, symptoms of depression and anxiety, fatigue, and personality traits were analyzed via ANOVA or Kruskal-Wallis tests, followed by post hoc comparisons. A discriminant analysis identified cognitive subtests that best differentiated the groups, while ANCOVA controlled for symptoms of depression and anxiety, and fatigue. Hierarchical cluster analysis (Ward’s method, Euclidean distance) was conducted on the entire sample to classify latent subgroups, with t-tests comparing cluster differences. All neuropsychological data were z-standardized based on the combined sample of 67 HC/CV participants, using their pooled mean and standard deviation (SD), and z-scores were interpreted according to conventional guidelines, with scores below − 1 SD considered indicative of cognitive impairment^31^.

## Results

### Demographics and clinical data

Overall, 54 PCS patients, 12 PV patients, 25 CV, and 42 HC were comparable in age, gender, and vaccination status. However, years of education differed significantly, with the Tukey post-hoc test revealing a difference between CV and HC. Significant differences were also observed in time since infection, with PV patients having the longest duration. During the acute infection, 2 PCS patients and 1 PV patient required hospitalization. The prevalence of lifetime mental disorders varied notably between groups, highest in the PCS group. A detailed overview of demographics and clinical data is provided in Table 1.

**Table 1:** Demographics and clinical data.

Characteristics	PCS (*N* = 54)		PV (*N* = 12)		HC (*N* = 42)		CV (*N* = 25)		Statistics^a^
	Mean/Median (SD)	*N* (%)	Mean/ Median (SD)	*N* (%)	Mean/ Median (SD)	*N* (%)	Mean/Median (SD)	*N* (%)	
**Age** (years)	43.52 (12.17)		34.58 (9.29)		43.40 (13.98)		38.08 (12.25)		F(3, 129) = 2.60, *p* =.055
**Female**		38 (70)		7 (58)		23 (55)		14 (56)	χ²(3) = 2.97, *p* =.396
**Male**		16 (30)		5 (42)		19 (45)		11 (44)	
**Education** (years)	14.41 (1.96)		15.21 (3.03)		**15.43** (2.32)		**13.84** (2.38)		**F(3**,** 129) = 3.15**, *p* =.027
**COVID-19 vaccination**									χ²(3) = 1.76, *p* =.624
vaccinated		51 (94)		10 (83)		39 (93)		23 (92)	
not vaccinated		3 (6)		2 (17)		3 (7)		2 (8)	
**Time since infection** (months)	20.74/21 (10.64)		37.42/**28** (29.29)		19.38/18.5 (11.99)		16.28/**15** (10.35)		**H(3) = 7.85**, *p* =.049
**Hospitalization during acute infection**		2 (4)		1 (8)		0 (0)		0 (0)	
**Lifetime mental disorders**		22 (43)		2 (17)		5 (12)		3 (12)	**χ²(12) = 29.07**, *p* =.004

Abbreviations: PCS = Post-COVID-19 syndrome. PV = Post-viral. HC = Healthy Controls. CV = Convalescents. Comparative analysis of demographic and clinical characteristics across the four subject groups (PCS, PV, HC, and CV).

^a^ Chi-square test, ANOVA or Kruskal-Wallis-Test for group comparison where appropriate; threshold for significant difference with *p* <.05. Group means/medians that significantly differed from each other are highlighted in bold.

### Cognitive performance during neuropsychological assessment

**Fig. 1:**
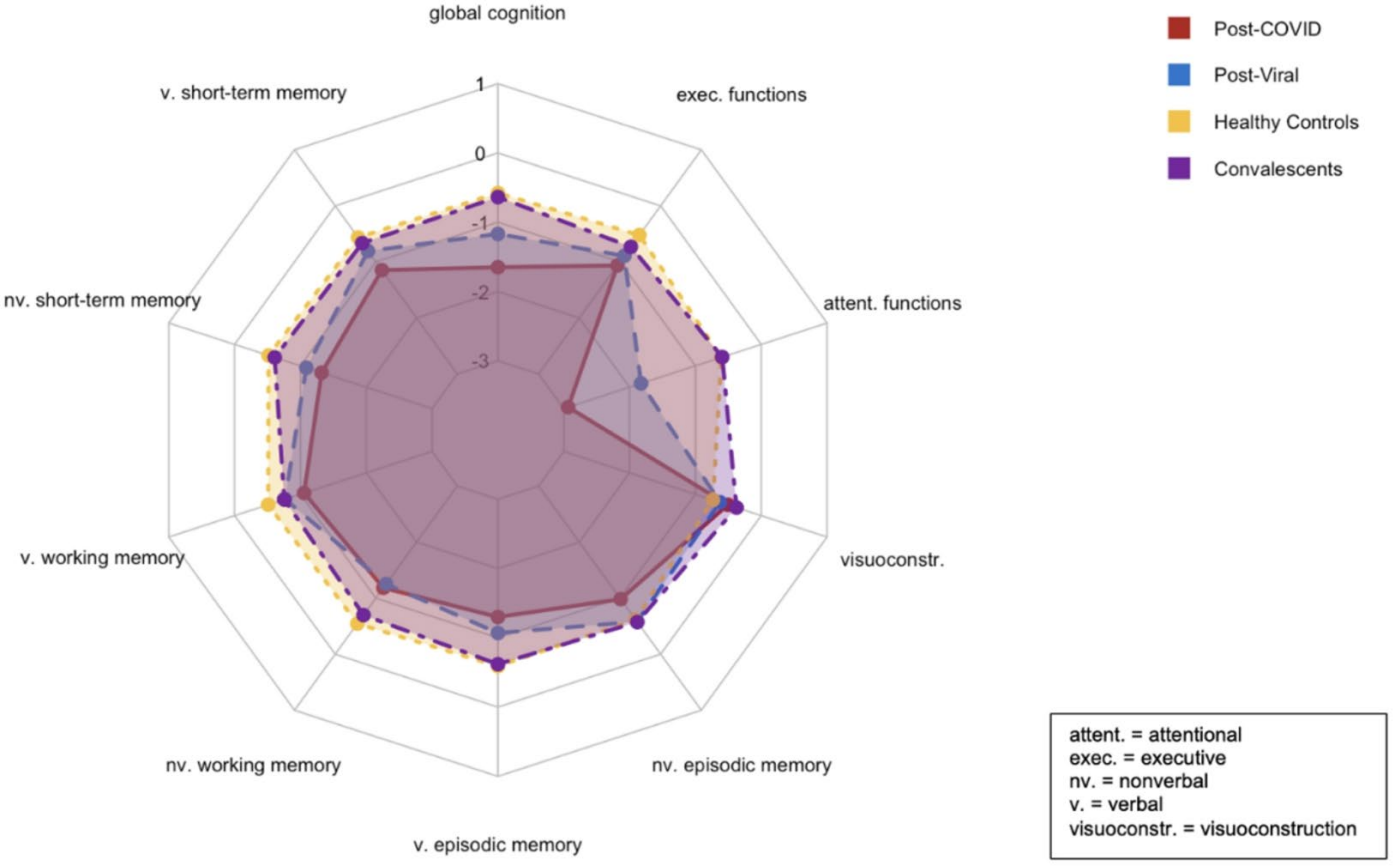
Between-group comparison of cognitive domains. Neuropsychological scores across cognitive domains (z-standardized). Composite scores for verbal episodic memory, attention, and executive functions were calculated by averaging z-standardized subscores. Global cognition was represented by a composite score summarizing all 20 cognitive subtests.

There were significant differences between groups across various cognitive domains and affective state (see Table 2; Fig. 1), with PCS patients performing worse than the HC group. In verbal short-term memory, the PCS group scored significantly lower in digit span forward tasks compared to HC (*ΔM*=−1.43, *p* =.003). Similarly, in nonverbal short-term memory, performance on the block-tapping test forward was worse in patients with PCS compared to HC (z=−3.49, *p* <.001) and CV (z=−3.11, *p* =.002). Working memory deficits were also prominent, with PCS patients performing worse than HC in digit span backward tasks (*ΔM*=−1.34, *p* =.003) and block-tapping test backward tasks (z=−3.03, *p* =.002). In verbal episodic memory (VLMT delayed recall), the PCS group scored significantly lower than HC (z=−3.69, *p* <.001) and CV (z=−2.91, *p* =.004). Attention deficits were evident, with poorer performance in both tonic (z=−4.32, *p* <.001) and phasic (z=−3.81, *p* <.001) alertness conditions in the PCS group compared to HC, reflecting impairments in sustained attention, arousal regulation, and the ability to rapidly allocate attentional resources in response to external stimuli. Additionally, reduced performance in divided attention, with impaired performance in both visual and auditory dual-task conditions were observed between PCS patients and HC (TAP divided attention auditory: z = 3.37, *p* <.001; visual: z = 4.91, *p* <.001) and CV, respectively (TAP divided attention visual: z = 4.15, *p* <.001). Furthermore, the PCS group exhibited significant reduced performance in inhibitory control compared to HC (TAP incompatibility: z = 3.62, *p* <.001) and CV (TAP incompatibility: z = 3.52, *p* <.001), as demonstrated by increased susceptibility to response incompatibility effects. Executive function scores were reduced, as indicated by reduced phonemic verbal fluency scores in the PCS group relative to HC (*ΔM*=−5.90, *p* <.001).

**Table 2:** Cognitive performance, fatigue and affective state across groups.

Cognitive domains Tests	PCS (*N* = 54)		PV (*N* = 12)		HC (*N* = 42)		CV (*N* = 25)		Statistics^a^
	Mean/ Median (SD)	*N* (%) impaired	Mean/Median (SD)	*N* (%) impaired	Mean/ Median (SD)	*N* (%) impaired	Mean/ Median (SD)	*N* (%) impaired	ANOVA/Kruskal-Wallis-Test
**Verbal short-term memory**									
Digit span forward	**6.50** (1.94)	22 (41)	7.33 (1.61)	2 (17)	**7.93** (1.94)	5 (12)	7.68 (2.02)	5 (20)	***F*(3**,** 129) = 4.87**, ***p*** **=.003**
**Nonverbal short-term memory**									
Block-Tapping-Test forward	**8** (2.13)	20 (37)	8 (1.75)	2 (17)	**9** (1.91)	4 (10)	**9** (1.38)	1 (4)	***H*(3) = 16.20**, ***p*** **=.001**
**Verbal working memory**									
Digit span backwards	**5.54** (1.51)	21 (39)	6.25 (2.34)	4 (33)	**6.88** (2.05)	8 (19)	6.28 (1.82)	6 (24)	***F*(3**,** 129) = 4.29**, ***p*** **=.006**
**Nonverbal working memory**									
Block-Tapping-Test backwards	**7** (2.24)	22 (41)	7 (1.82)	5 (42)	**9** (1.57)	3 (7)	8 (1.83)	6 (24)	***H*(3) = 12.71**, ***p*** **=.005**
**Verbal episodic memory**									
VLMT total	**52.50** (11.91)	12 (22)	55.00 (10.72)	2 (17)	**58.67** (8.75)	1 (2)	**57.52** (7.28)	2 (8)	***F*(3**,** 129) = 3.29**, ***p*** **=.023**
VLMT delayed recall	11.50 (3.15)	16 (30)	12 (3.42)	5 (42)	14 (2.41)	3 (7)	13 (1.88)	0 (0)	*H*(3) = 16.59, *p* <.001
VLMT recognition	**14** (1.53)	13 (24)	14 (1.44)	2 (17)	**15** (0.86)	3 (7)	**15** (0.77)	1 (4)	***H*(3) = 10.09**, ***p*** **=.018**
**Visuoconstruction**									
Rey-Osterrieth Complex Figure Test (copy)	36 (1.00)	2 (4)	36 (1.30)	1 (8)	36 (1.30)	3 (7)	36 (0.56)	0 (0)	*H*(3) = 1.64, *p* =.650
**Nonverbal episodic memory**									
Rey-Osterrieth Complex Figure Test (delayed recall)	21.76 (6.85)	9 (17)	25.04 (6.72)	3 (25)	24.74 (6.84)	5 (12)	25.08 (6.05)	2 (8)	*F*(3, 129) = 2.36, *p* =.075
**Attentional functions**									
TAP Alertness (tonic)	**283** (295.79)	28 (52)	291.50 (114.02)	9 (75)	**231** (30.44)	4 (10)	**227** (29.75)	2 (8)	***H*(3) = 32.10**, ***p*** **<.001**
TAP Alertness (phasic)	**281** (263.03)	32 (59)	280 (59.76)	9 (75)	**235.50** (34.08)	9 (21)	**231** (29.76)	3 (12)	***H*(3) = 24.26**, ***p*** **<.001**
TAP Divided Attention (auditory)	**662** (287.68)	32 (59)	686 (116.31)	9 (75)	**567.50** (111.60)	15 (36)	**622** (83.48)	13 (52)	***H*(3) = 14.39**, ***p*** **=.002**
TAP Divided Attention (visual)	**834** (197.62)	14 (26)	788 (114.50)	3 (25)	**726.50** (83.00)	2 (5)	**727** (84.53)	1 (4)	***H*(3) = 32.90**, ***p*** **<.001**
TAP Incompatibility	546.50 (284.05)	26 (48)	517 (147.30)	6 (50)	444.50 (77.03)	6 (14)	435 (71.38)	3 (12)	*H*(3) = 20.08, *p* <.001
SDMT	**50.28** (12.05)	22 (41)	54.00 (11.91)	6 (50)	**60.17** (8.85)	3 (7)	**61.12** (8.95)	2 (8)	***F*(3**,** 129) = 9.56**, ***p*** **<.001**
**Executive functions**									
Phonemic verbal fluency (RWT S)	**19.39** (5.75)	28 (52)	22.75 (10.84)	8 (67)	**25.29** (7.60)	10 (24)	**23.72** (6.64)	11 (44)	***F*(3**,** 129) = 5.87**, ***p*** **<.001**
Semantic verbal fluency (RWT food)	**34.76** (8.74)	19 (35)	38.00 (11.93)	5 (42)	**41.36** (10.25)	9 (21)	**40.04** (12.43)	6 (24)	***F*(3**,** 129) = 3.60**, ***p*** **=.015**
Semantic verbal fluency (RWT animals)	**32.70** (9.94)	28 (52)	34.33 (8.13)	8 (67)	**40.90** (11.07)	10 (24)	**37.76** (10.08)	11 (44)	***F*(3**,** 129) = 4.41**, ***p*** **=.002**
Phonemic category change (RWT G-R)	**17.31** (5.12)	29 (54)	19.83 (3.93)	6 (50)	**22.76** (7.08)	11 (26)	20.76 (5.40)	8 (32)	***F*(3**,** 129) = 7.21**, ***p*** **<.001**
Semantic category change (RWT sports-fruits)	21.56 (6.07)	12 (22)	20.42 (6.26)	7 (58)	23.38 (5.61)	4 (10)	21.56 (3.31)	4 (16)	*F*(3, 129) = 1.37, *p* =.255
**Cognitive Fatigue (FSMC)**	**43** (5.22)		40 (10.78)		**14** (6.00)		**17** (7.00)		***H*(3) = 95.34**, ***p*** **<.001**
Mild		0 (0)		0 (0)		7 (17)		6 (24)	
Moderate		4 (7)		2 (17)		2 (5)		3 (12)	
Severe		50 (93)		8 (67)		0 (0)		0 (0)	
Motor Fatigue (FSMC)	42.22 (5.06)		36.00 (9.19)		15.62 (5.98)		15.92 (5.42)		*F*(3, 129) = 210.06, *p* <.001
Mild		0 (0)		0 (0)		5 (12)		7 (28)	
Moderate		2 (4)		2 (17)		3 (7)		0 (0)	
Severe		52 (96)		9 (75)		0 (0)		0 (0)	
**Depressive symptoms ** **(PHQ-9)**	**12.70** (4.69)		9.42 (3.15)		**3.98** (3.63)		**4.80** (2.90)		***F*(3**,** 129) = 45.86**, ***p*** **<.001**
Mild		23 (43)		6 (50)		1 (2)		0 (0)	
Moderate		16 (30)		0 (0)		2 (5)		0 (0)	
Severe		4 (7)		0 (0)		0 (0)		0 (0)	
**Anxiety symptoms (GAD-2)**	**2** (1.60)		2 (1.67)		**1** (1.04)		**1** (0.79)		***H*(3) = 21.83**, ***p*** **<.001**
Increased anxiety symptoms		20 (37)		1 (8)		2 (5)		1 (4)	
none		34(63)		11(92)		40(95)		24(96)	
**Personality traits**									
Neuroticism (NEO-FFI-30)	**1.66** (0.92)		1.38 (0.74)		**1.07** (0.83)		**1.10** (0.65)		***F*(3**,** 129) = 4.81**, ***p*** **=.003**
Extraversion (NEO-FFI-30)	**1.88** (0.79)		2.03 (0.48)		**2.42** (0.64)		**2.37** (0.70)		***F*(3**,** 129) = 5.62**, ***p*** **=.001**
Openness (NEO-FFI-30)	2.30 (0.86)		2.64 (0.70)		2.37 (0.75)		2.35 (1.12)		*F*(3, 129) = 0.50, *p* =.684
Agreeableness (NEO-FFI-30)	3.21 (0.56)		3.26 (0.59)		3.17 (0.65)		3.03 (0.63)		*F*(3, 129) = 0.58, *p* =.627
Conscientiousness (NEO-FFI-30)	3.10 (0.62)		3.35 (0.76)		3.31 (0.65)		3.09 (0.67)		*F*(3, 129) = 1.24, *p* =.298

Abbreviations: PCS = Post-COVID-19 syndrome. PV = Post-viral. HC = Healthy Controls. CV = Convalescents. VLMT = Verbal Learning Memory Test. TAP = Testbatterie zur Aufmerksamkeitsprüfung. SDMT = Symbol Digit Modalities Test. RWT = Regensburger Wortflüssigkeitstest (German Version of Verbal Fluency). FSMC = Fatigue Scale for Motor and Cognitive Functions. PHQ-9 = Patient Health Questionnaire-9. GAD-2 = Generalized Anxiety Disorder-2. NEO-FFI-30 = NEO-Five-Factor Inventory (30-Item-Short-Version). ^a^ANOVA or Kruskal-Wallis-Test for group comparison where appropriate; threshold for significant difference with *p* <.05. For variables that did not meet the assumption of homogeneity of variances, the median is reported instead of the mean. Group means/medians that significantly differed from each other are highlighted in bold.

### Affective state and fatigue

Patient-reported outcome measures demonstrated significant differences between groups, with scores of depressive symptoms (PHQ-9) being significantly higher in PCS patients compared to HC (*ΔM* = 8.73, *p* <.001) and CV (*ΔM* = 7.90, *p* <.001). Anxiety was more prevalent in PCS (37%) compared to HC (4.8%).

The most pronounced effects were observed in cognitive and motor fatigue (FSMC). PCS patients reported significantly higher cognitive fatigue compared to HC (z = 8.70, *p* <.001) and CV (z = 7.01, *p* <.001), with severe cognitive fatigue affecting 92.6% of the PCS group (0% in HC). Similarly, motor fatigue was markedly higher in PCS than in HC (*ΔM* = 26.60, *p* <.001) and CV (*ΔM* = 26.30, *p* <.001), with 96.3% of PCS patients experiencing severe motor fatigue compared to none in HC.

### Definition of most discriminant cognitive domains

To investigate whether the observed group differences also manifest at the individual level, we first conducted a discriminant analysis and identified the tests that best differentiated between groups.

The highest discrimination power was shown by the SDMT (*r* =.61, *β* = 0.35), the TAP subtests Alertness (tonic, *r*=-.60, *β*=−0.57 and phasic, *r*=-.56, *β* = 0.54), Divided Attention (visual, *r*=-.60, *β*=−0.19), and Incompatibility (*r*=-.58, *β*=−0.19), the Block-Tapping Test forward (*r* =.52, *β* = 0.28), the VLMT Delayed Recall (*r* =.51, *β* = 0.38), the Phonemic Verbal Fluency (RWT letter “S”) (*r* =.52, *β* = 0.27), and the Phonemic Category Change (RWT letter “G-R”) (*r* =.51, *β* = 0.24). The first discriminant function accounted for 75.4% of the variance (eigenvalue = 0.593, canonical correlation = 0.610) but narrowly missed the threshold for statistical significance (*Λ* = 0.522, *χ²(*60) = 77.351, *p* =.065). Subsequently, separate ANCOVAs were conducted on cognitive tests that met the discriminant coefficient cut-off (*r* ≥.5), controlling for symptoms of depression and anxiety, motor fatigue, cognitive fatigue, and group membership. Results indicated that none of these covariates had a significant effect on the observed differences between groups in the cognitive tests (Block-Tapping Test forward, VLMT Delayed Recall, TAP Alertness tonic and phasic, TAP Divided Attention visual, TAP Incompatibility, SDMT, RWT letter “S” and “G-R”; all F≥0.01, *p* >.05, *η²*>0.00).

### Classification of subgroups

**Fig. 2:**
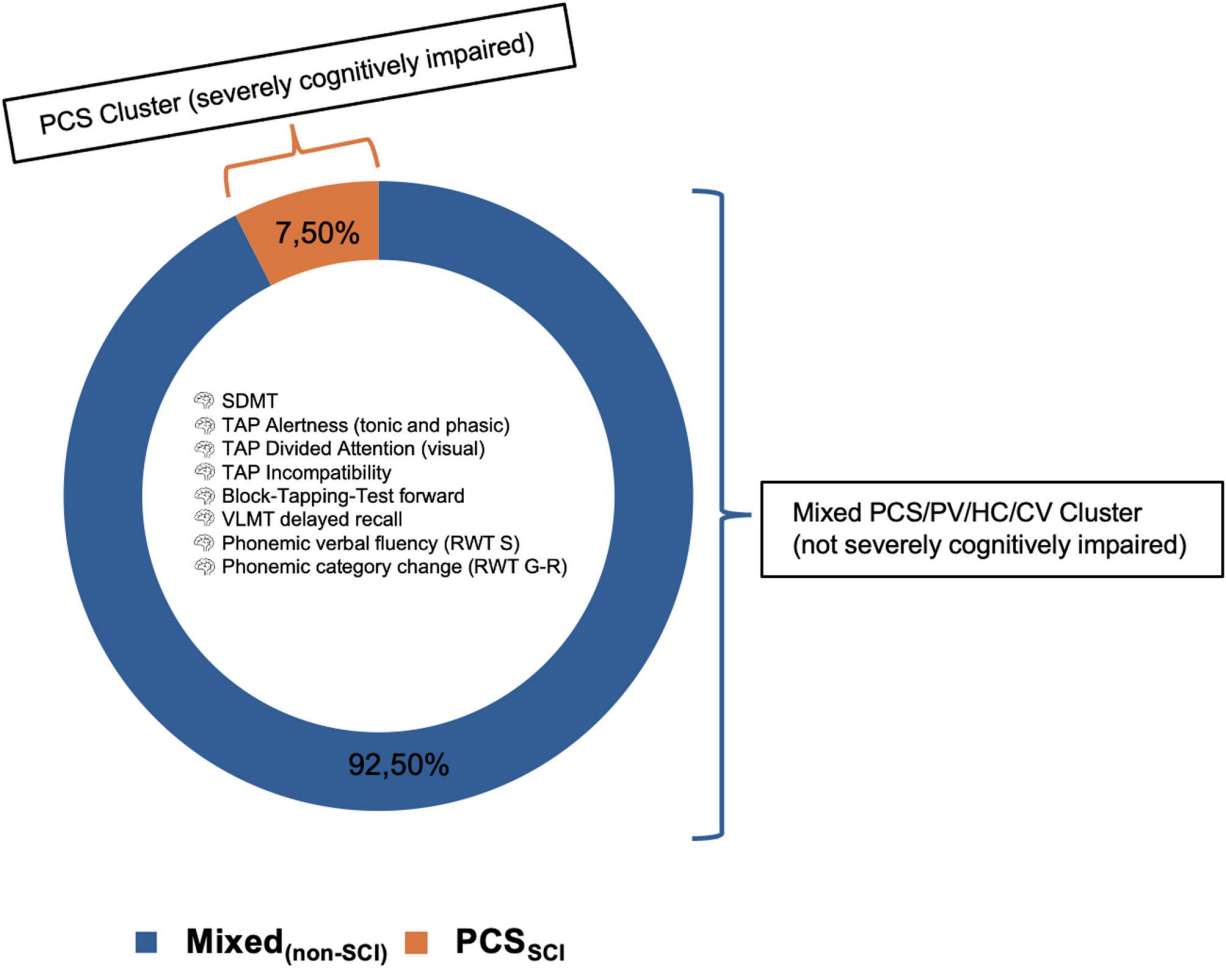
Cognitive Performance-Based Clustering: Severe vs. Mild Subgroup. Key cognitive performance measures reveal two distinct clusters with a cluster (orange) of pure severely cognitively impaired PCS patients (PCS_SCI_), and a mixed cluster (blue) with a mixed sample of PCS, PV, HC, and CV with no or only moderate objective cognitive impairment, indicating average range of cognitive dysfunction within the study population (Mixed_(non−SCI)_). Hierarchical cluster analysis with Ward’s method and Euclidean distance; threshold for significant difference with *p* <.05.

Using the cognitive key discriminators mentioned above as the basis for a data-driven group assignment, a hierarchical cluster analysis according to the Ward method was calculated across all patients, identifying two clusters: a heterogeneous Cluster 1 (*n* = 123), including unimpaired to moderately impaired PCS, PV, HC, CV subjects, indicating average to mild range of cognitive dysfunction within the study population (Mixed_(non-SCI)_), and a homogeneous Cluster 2 (*n* = 10), composed exclusively of PCS patients with severe objective cognitive impairment (PCS_SCI_; see Fig. 2 and Supplementary Fig. S1).

### Characteristics of PCS_SCI_ compared to Mixed_(non−SCI)_

Subgroups of PCS_SCI_ and Mixed_(non-SCI)_ significantly differed in psychiatric history and fatigue, but demographic factors were comparable. The mean age (M_Mixed(non-SCI)_ = 41.37 years vs. M_PCS SCI_=45.10 years, t(131)=−0.88, *p* =.378), education level (M_Mixed(non-SCI)_ = 14.69 years, M _PCS SCI_=14.80 years; t(131)=−0.15, *p* =.883), and time since infection (M_Mixed(non-SCI)_ = 21.02 months vs. M_PCS SCI_=20.4 months, t(131) = 0.13, *p* =.896) showed no significant differences between subgroups.

In contrast, mental disorders and fatigue differed substantially. Lifetime mental disorders were more prevalent in PCS_SCI_ (60%) compared to Mixed_(non-SCI)_ (22%) (*χ²(*4) = 14.84, *p* =.005). Current symptoms of depression were also more common in PCS_SCI_ (60% vs. 34.9%) (t(131)=−3.10, *p* =.002). Anxiety symptoms showed the same frequency between subgroups (Mixed_(non-SCI)_: 17.9%, PCS_SCI_: 20%) (t(131)=−0.54, *p* =.592).

Cognitive and motor fatigue were significantly more severe in PCS_SCI_ participants. Severe cognitive fatigue was prevalent in all PCS_SCI_ participants (100%) vs. 39% in Mixed_(non-SCI)_ (t(54.57)=−10.53, *p* <.001), and severe motor fatigue was reported by 100% of PCS_SCI_ participants, compared to 41.5% in Mixed_(non-SCI)_ participants (t(71.67)=−10.95, *p* <.001).

No significant differences were found in personality traits between PCS_SCI_ and Mixed_(non-SCI)_: neuroticism (t(131)=−0.52, *p* =.603), extraversion (t(131) = 0.60, *p* =.548), openness (t(131) = 0.09, *p* =.927), agreeableness (t(131)=−1.38, *p* =.170), and conscientiousness (t(20.52)=−1.30, *p* =.208).

## Discussion

Within this unique clinical cohort of subjectively impaired PCS subjects after COVID-19, we hereby defined a specific 7.5% subgroup of severely cognitively impaired subjects (PCS_SCI_). They provided a distinctly different cognitive profile on key cognitive domains, including attentional, memory, and executive function, based on cognitive tests for which group differences were not significantly influenced by depression, anxiety, and fatigue. This underscores the dominant role of deficits in these cognitive domains in PCS, aligning with recent literature^2–4,32,33^. However, in most studies on PCS patients, in-depth neurological assessment has not yielded pathological findings, suggesting that cognitive symptoms may stem from psychosomatic mechanisms rather than direct neural damage^32^. Accordingly, within the hereby presented cluster-based analysis, a diverse group of up to moderately impaired subjects with regard to cognitive profile (Mixed_(non-SCI)_) was revealed, including PCS patients with subjective complaints whose objective cognitive performance was indistinguishable from that of post-viral patients, healthy controls, or convalescents. Most interestingly, PV subjects similarly to PCS subjects subjectively complained about cognitive impairment, whereas the other subjects in the Mixed_(non-SCI)_ cluster didn’t report on subjective cognitive impairment (e.g., anosognosia^34^. But PCS patients in the average group (and PV patients) did not exhibit significantly higher objective cognitive impairment, suggesting that subjective complaints may not necessarily indicate objective deficits. As all subjects included in the study had experienced viral infections of different etiology (COVID-19 (PCS, CV, and HC) or Epstein-Barr virus infection (PV)), this profile of up to moderate cognitive impairment may resemble a general post-viral phenomenon, known to prevail for months to years in other post-viral conditions e.g. Epstein-Barr virus infection^35^. And this up to moderate cognitive impairment may exist unnoticed by the subject him-/herself as the hereby included CV patients attended our outpatient clinic due to physical symptoms but not cognitive symptoms but presented with a similar cognitive profile as the PCS subjects. In post viral state, fatigue, attentional biases, or affective state may contribute to perceived impairments without reflecting substantial neurocognitive decline^15,18,36^. The variability in Mixed_(non-SCI)_ subjects may reflect natural variance in cognitive performance of no to moderate cognitive impairments in post viral infection state rather than a distinct pathological pattern of cognitive decline due to COVID-19 in specific^11,37,38^.

Moreover, our results support the notion that PCS-related cognitive impairments are not merely artifacts of distress^10,39,40^. Specifically, PCS patients exhibited deficits in processing speed and executive functioning, independent of affective state, which is consistent with existing literature^32,41,42^. Most interestingly, the small subset (*n* = 10) of PCS_SCI_ subjects with severe objective cognitive impairment displayed pronounced depressive symptoms. But also within this subgroup, cognitive performance was not simply explained by affective state or fatigue. Thus, cognitive performance and distress (depression and anxiety) may evolve separately in PCS patients but may still be interrelated. Cause and effect may indistinguishable, as cognitive impairment may interfere with affective state and vice versa. Interrelation with neuroinflammation, dysregulated stress responses, or autonomic dysfunction may additionally trigger a cascade of imbalance^9^. The biopsychosocial model offers a useful framework to understand these interactions, emphasizing that PCS symptoms may arise from an interplay of biological, psychological, and social factors^38,43^. In functional somatic syndrome (FSS) patients, negative affect can trigger physiological symptoms via the activation of somatosensory and nociceptive brain patterns^33^. In line, high somatization scores found in PCS patients suggest a strong psychosomatic component in symptom expression^32^. Also, lifetime vulnerability pattern for psychiatric conditions, such as symptoms of depression and anxiety, have been identified as risk factors for developing Long-COVID syndrome^38^. However, no significant differences in personality traits were found between PCS_SCI_ and Mixed_(non-SCI)_, indicating that stable dispositional factors such as neuroticism or conscientiousness did not significantly influence the observed cognitive or affective differences. This also suggests that PCS-related symptoms might be more closely associated with state-dependent psychosomatic mechanisms rather than long-lasting personality traits. The traditional medical view focusing primarily on the pathophysiology of a disease has struggled to address these complexities, often leaving PCS patients feeling dismissed when no clear pathology is identified^44,45^. Yet, persistent physical symptoms may be exacerbated by stigma and distress when patients feel disbelieved or misunderstood^43^. The same may be true for PCS patients.

While PCS is often linked to distress, particularly depression and anxiety, our findings reveal a more complex and heterogeneous picture, particularly regarding cognitive functioning. A subgroup of subjects may display a profile of severe cognitive performance, which may need special attention in clinical care. Whether this severe cognitive pathology is triggered by a more pronounced biophysical cascade, such as prolonged neuroinflammation – as has been suggested in post-viral syndromes and other inflammatory conditions^46^ – is purely speculative but needs to be addressed in future research.

### Limitations

A key limitation of our study is the unequal sample sizes across groups, with particularly small numbers in the post-viral group of other etiology and the convalescent group. This imbalance may limit generalizability and calls for further research with larger, more balanced samples. To our knowledge, this is the first study including post-viral subjects with either subjective cognitive (PCS, PV), physical (CV), or no complaints (HC), disentangling the misconception that subjective reports reflect objective deficits. This unique cohort suggests a general post-viral cognitive profile, subdivided into: (1) mild to moderately impaired individuals (Mixed_(non-SCI)_), with or without complaints, and (2) a severely affected Post-COVID subgroup (PCS_SCI_) with subjective complaints and major objective impairments. The latter requires further study. As neuropsychological data were z-standardized based on the combined HC/CV group, this approach may have introduced bias in group comparisons; however, it ensured consistent and demographically appropriate scaling across all measures. Furthermore, as this study involved multiple group comparisons across a broad neuropsychological test battery, no formal correction for multiple testing (e.g., False Discovery Rate) was applied. This increases the risk of Type I errors, which should be considered when interpreting the findings. Additionally, no formal performance validity tests (PVTs) were administered, and although all participants were assessed under standardized supervision without signs of insufficient effort, the absence of embedded or standalone PVTs limits our ability to fully rule out potential effects of reduced task engagement on neuropsychological outcomes^47^.

It is also important to acknowledge that information on lifetime mental disorders relied on patient self-reports only, which may not fully capture all past conditions, as some patients might minimize symptoms or have never sought professional consultation.

## Conclusions

Our study provides evidence that PCS patients display a heterogeneous (neuro)psychological profile, comparable to other post-viral subjects. A small distinct subgroup with severe objective cognitive impairment in PCS patients (PCS_SCI_) shows heightened psychological vulnerability, warranting further investigation. Given their severity of objective cognitive impairment and distress, a targeted multimodal treatment, combining especially neuropsychological and psychotherapeutic interventions may be essential for supporting cognitive and emotional well-being. These findings highlight the need for comprehensive clinical and scientific assessments in PCS, as broad generalizations may overlook clinically relevant subgroups.

## Supplementary Information

Below is the link to the electronic supplementary material.

Supplementary Material 1

## Data Availability

Anonymized data will be shared upon reasonable request from a qualified investigator (corresponding author: dorothee.lule@uni-ulm.de).
