# Correction: Evidence for a severe cognitive subgroup in a comprehensive neuropsychological Post-COVID-19 syndrome classification

**DOI:** 10.1038/s41598-025-33148-7

**Published:** 2025-12-30

**Authors:** Luisa T. Balz, Deborah K. Erhart, Ingo Uttner, Dorothée E. Lulé, Hayrettin Tumani

**Affiliations:** Department of Neurology, Faculty of Medicine, Ulm University Oberer Eselsberg, 45-89071 Ulm, Germany

Correction to: *Scientific Reports* 10.1038/s41598-025-25453-y, published online 18 November 2025

The original version of this Article contained errors in the Figure 1 and 2 legends, where the legends were part of the main text, consequently, Figure 1 legend:

“Between-group comparison of cognitive domains.”

now reads:

“Between-group comparison of cognitive domains. Neuropsychological scores across cognitive domains (z-standardized). Composite scores for verbal episodic memory, attention, and executive functions were calculated by averaging z-standardized subscores. Global cognition was represented by a composite score summarizing all 20 cognitive subtests.”

Figure 2 legend,

“Cognitive Performance-Based Clustering: Severe vs. Mild Subgroup.”

now reads:

“Cognitive Performance-Based Clustering: Severe vs. Mild Subgroup. Key cognitive performance measures reveal two distinct clusters with a cluster (orange) of pure severely cognitively impaired PCS patients (PCS_SCI_), and a mixed cluster (blue) with a mixed sample of PCS, PV, HC, and CV with no or only moderate objective cognitive impairment, indicating average range of cognitive dysfunction within the study population (Mixed_(non−SCI)_). Hierarchical cluster analysis with Ward’s method and Euclidean distance; threshold for significant difference with *p* <.05.”

The original Article has been corrected.

