# Supplementary material for: Evidence for a severe cognitive subgroup in a comprehensive neuropsychological Post-COVID-19 syndrome classification
Source: Sci Rep. 2025 Nov 18;15:40368. doi: 10.1038/s41598-025-25453-y (PMC12627843; doi:10.1038/s41598-025-25453-y)
Supplement: Supplementary file 1 — Supplementary Material 1 [file 41598_2025_25453_MOESM1_ESM.pdf]

Supplementary Material

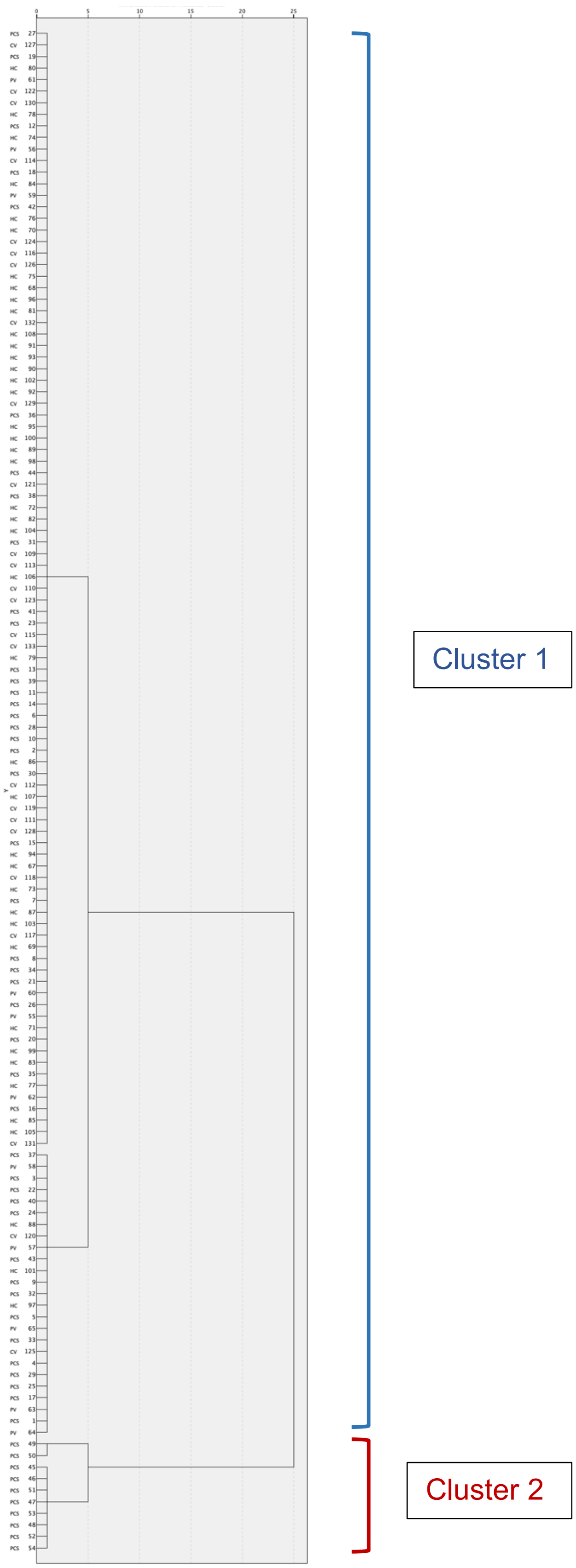

Fig. S1: Dendrogram of cluster analysis with selected cognitive parameters. Two

distinct clusters were identified: Cluster 1 (blue), which includes the majority of participants (MCI_{ALL}), and Cluster 2 (red), a smaller subgroup representing patients with PCS with severe cognitive impairment (SCI_{PCS}). Clustering was conducted using Ward's method and Euclidean distance.
